# Beyond the snapshot: identification of the timeless, enduring indicator microbiome informing soil fertility and crop production in alkaline soils

**DOI:** 10.1186/s40793-022-00420-6

**Published:** 2022-05-12

**Authors:** Jianwei Zhang, Jan Dolfing, Wenjing Liu, Ruirui Chen, Jiabao Zhang, Xiangui Lin, Youzhi Feng

**Affiliations:** 1 State Key Laboratory of Soil and Sustainable Agriculture, Institute of Soil Science, Chinese Academy of Sciences, Nanjing, 210008 People’s Republic of China; 2 University of Chinese Academy of Sciences, Beijing, 100049 People’s Republic of China; 3 Faculty of Engineering and Environment, Northumbria University, Newcastle Upon Tyne, UK

**Keywords:** Soil microorganisms, Soil fertility, Sustainable agroecosystem, Archived soils, Fertilization

## Abstract

**Background:**

Microorganisms are known to be important drivers of biogeochemical cycling in soil and hence could act as a proxy informing on soil conditions in ecosystems. Identifying microbiomes indicative for soil fertility and crop production is important for the development of the next generation of sustainable agriculture. Earlier researches based on one-time sampling have revealed various indicator microbiomes for distinct agroecosystems and agricultural practices as well as their importance in supporting sustainable productivity. However, these microbiomes were based on a mere snapshot of a dynamic microbial community which is subject to significant changes over time. Currently true indicator microbiomes based on long-term, multi-annual monitoring are not available.

**Results:**

Here, using samples from a continuous 20-year field study encompassing seven fertilization strategies, we identified the indicator microbiomes ecophysiologically informing on soil fertility and crop production in the main agricultural production base in China. Among a total of 29,184 phylotypes in 588 samples, we retrieved a streamlined consortium including 2% of phylotypes that were ubiquitously present in alkaline soils while contributing up to half of the whole community; many of them were associated with carbon and nutrient cycling. Furthermore, these phylotypes formed two opposite microbiomes. One indicator microbiome dominated by *Bacillus asahii*, characterized by specific functional traits related to organic matter decomposition, was mainly observed in organic farming and closely associated with higher soil fertility and crop production. The counter microbiome, characterized by known nitrifiers (e.g., *Nitrosospira multiformis*) as well as plant pathogens (e.g., *Bacillus anthracis*) was observed in nutrient-deficit chemical fertilizations. Both microbiomes are expected to be valuable indictors in informing crop yield and soil fertility, regulated by agricultural management.

**Conclusions:**

Our findings based on this more than 2-decade long field study demonstrate the exciting potential of employing microorganisms and maximizing their functions in future agroecosystems. Our results report a “most-wanted” or “most-unwanted” list of microbial phylotypes that are ready candidates to guide the development of sustainable agriculture in alkaline soils.

**Supplementary Information:**

The online version contains supplementary material available at 10.1186/s40793-022-00420-6.

## Background

Soil microorganisms with their incredible genetic and ecophysiological diversity play essential roles in regulating multiple ecosystem services, including nutrient cycling, organic matter decomposition, plant productivity, and pathogen control [1–3]. However, the immense diversity of soil bacterial communities has stymied efforts to characterize functional taxa and/or consortia [4]. Recent research suggests alternative strategies to focus on a relatively small subset of phylotypes instead of exhaustively screening that overwhelming diversity [5–7]. These refined phylotypes are indispensably present in communities with a high frequency and they self-assemble into consortia that could characterize divergent environmental conditions (hereafter termed indicator microbiomes) [4, 8]. Such streamlined but ubiquitous microbiomes will be fruitful targets for genomic and cultivation-based efforts to improve our understanding of soil microbes and their contributions to ecosystem functioning [4, 6–8].

Agroecosystems are shaped by entangled webs of interactions among diversified microorganisms. Species composition and interactions in microbial communities determine the ecological functions and services they provide and hence they play a pivotal role in maintaining ecosystem sustainability [9]. Harnessing microorganisms and maximizing their functions in agroecosystems offers one of the most promising long-term solutions to meet the integral demand for simultaneously promoting crop production and environmental sustainability [6, 10]. Phylogenetically distinct soil microbes inherently exhibit divergent niche preferences, which in turn mirror different environmental conditions [4, 8], e.g., a high fertility soil with high crop production, and vice versa.

Fertilization is one of the most impactful human manipulations of agroecosystems [11], where divergent farming systems supported distinct microbiomes depending on and indicative of the conditions in the agroecosystem concerned [12, 13]. Divergent fertilization practices provide us with the opportunity to unravel the indicator microbiome associated with nutrient cycling, for example, nitrogen addition management shifting the microbiome related to N cycling [12] and organic matter amendment linking the microbiome associated with the decomposition of external organic matter [13, 14]. In this context, a systematic long-term field experiment including the frequent application of multiple fertilization types could give us a general indicator microbiome associated with soil nutrient cycling and hence crop production [15–17].

Without strong external perturbation, soil microbial community composition can vary on scales from days to years due to stochastic drift and speciation in combination with ongoing dispersal processes [18–20]. Given this situation, microbial temporal patterns cannot necessarily be generalized even if soils are exposed to the same climatic conditions [21], which questions the existence of consistent indicator microbiomes that could resist temporal variability [18, 21]. On the other hand, the situation is different when deterministic influences from strong external perturbations exceed the inherent stochastic temporal variability in the microbial community [21, 22]. For example, fertilization is a strong environmental filter that significantly shapes the environment, and hence shifts the microbial community therein [6, 23, 24]. In this respect, we hypothesized that specific indicator microbiomes consistently emerge during long-term distinct fertilization strategies, which can be indicative of soil fertility and crop yield. Indeed, such a comprehensive temporal analysis of indicator microbiomes would transcend noisy information from typical snapshot studies [7, 21] and is essential for refinement of the identification and application of indicator microbiomes that are critical for crop yield and soil fertility.

Here, we performed a retrospective analysis of a 20-year long fertilization experiment encompassing 588 samples from seven contrasting fertilizer treatments, representing trajectories of farming systems from conventional to organic and from nutrient-deficiency to nutrient-balance. These unique samples enable us to investigate the existence, composition and structure of indicator microbiomes across a decadal scale that are closely related to soil fertility and crop production under various management practices. This long-term field experiment was conducted on the Northern China Plain, where winter wheat-summer maize rotation is the dominant cropping system and crop production is primarily constrained by low indigenous fertility of the alkaline soil [14]. Approximately 30% of arable land in the world suffers from alkaline stress limiting crop production [25], and this challenge is compounded by global aridification [26, 27]. Wheat and maize are among the most widely grown commodity crops around the world, accounting for a considerable amount of cropland arable area, yielding non-meat calories and animal feed [28]. In this respect, the identified indicator microbiome for soil fertility and crop yield from this study, although generated from a specific sampling site, could provide critical information for dryland ecosystems on a global scale that suffer from soil alkalinity.

## Material and methods

### Study sites and sample collection

Soil samples were collected from a long-term nutrient fertilization experiment at the Fengqiu Ecological Experimental Station affiliated to Institute of Soil Science, Chinese Academy of Sciences (Kaifeng City, Henan Province, 35° 00′ N, 114° 24′ E). The soil is classified as Aquic Inceptisol (a calcareous fluvoaquic soil), a typical alkaline soil in the North China Plain derived from alluvial sediments of the Yellow River. The soil pH is approximately 8.65, with total organic matter at 5.83 g/kg, total N at 0.45 g/kg, total P at 0.5 g/kg, and total K at 18.5 g/kg at the beginning of the experiment [14]. The crop succession was winter wheat (*Triticum aestivum L.*) and summer maize (*Zea mays L.*). This experiment was established in 1989 and consisted of seven fertilization regimes with four replicates in randomized plots as follows [14]: (1) OM: organic manure (supplemented with P and K as chemical fertilizers for the same amount of nutrients as in the other treatments); (2) NPKM: half organic manure plus chemical fertilizer NPK; (3) NPK: balanced chemical fertilizer NPK; (4) NP: chemical fertilizer NP; (5) PK: chemical fertilizer PK; (6) NK: chemical fertilizer NK; and (7) Control: no fertilization. The detailed experimental design and fertilization regimes have been documented in previous work [29, 30], and could be referred in Additional file 2: Table S1. Briefly, each plot measured 9.5 * 5 m^2^. All phosphorus (calcium superphosphate), potassium (potassium sulfate) and organic manure fertilizers (mixture of wheat straw, oil rapeseed cake, and cottonseed cake in a ratio of 100:40:45) were applied as basal fertilizers, whereas N (urea) was added in two applications as both the basal (60 kg ha^−1^ N for maize and 90 kg ha^−1^ N for wheat, respectively) and supplementary fertilizer (90 kg ha^−1^ N for maize and 60 kg ha^−1^ N for wheat, respectively). Crop yields were recorded annually and were generally increased under fertilizations except for those under N- and P-deficient fertilizers (PK and NK, respectively) and unfertilized Control (Additional file 1: Fig. S1). Soil samples were collected annually after the harvest of summer maize (typically in October), air-dried and archived in stoppered glass bottles. In 2019, we retrieved archived soil samples from 1989 to 2009, yielding 588 samples (21 years × 7 fertilization regimes × 4 replicates) in total. Soil C and N were measured with a LECO analyzer, and soil P and K were measured by colorimetry.

### DNA sequencing and bacterial diversity analysis

Genomic DNA was extracted from archived soils using the FastDNA Spin Kit for Soil (MP Biochemicals, Solon, OH, USA), with some modifications to the manufacturer protocol. Briefly, soils of around 500 mg (as dry weight) were weighed into prepared centrifuge tubes (prechilled to 4 °C) containing buffer and extraction glass beads. The samples were incubated for 30 min for rehydration, and then cells were lysed in a FastPrep-24 Homogenizer (6.0 setting, 40 s; MP Biochemicals, USA). The samples were then incubated at 70 °C for 10 min to aid the lysis of Gram-positive bacteria, and briefly (~ 10 s) re-shaken in the homogenizer. The extracted soil DNA was dissolved in 50 μl of TE buffer, quantified by a spectrophotometer and stored at − 20 °C (long-term storage in − 80 °C). Despite air-drying and long-term storage, high-quality DNA was recovered from the soils archived for decades (260/280 ~ 1.8 ([31]). We have been previously published based on DGGE analyses of these samples [14], which confirmed the systematic difference between samples from divergent farming systems and reported that *Bacillus asahii* bloomed from 2 to 4 years onward under organic fertilization. In this study, Illumina high-throughput sequencing platform was used to dissect more sophisticated information on the bacterial community composition. For each soil sample, the primer set 519F/907R [32] was used to amplify approximately 400 bp of V4–V5 region of bacterial 16S rRNA gene fragments. The 5-bp length oligonucleotide sequences were fused to the forward primer to distinguish individual samples. PCR was carried out in 50 μl mixtures with the following components (final concentrations): 200 µM dNTPs; 0.6 μM forward and reverse primers; 2 units/50 ul Taq DNA polymerase (TaKaRa, Japan); around 1 ng/μl template DNA; add sterile water to 50 ul. Twenty-five cycles (95 °C for 45 s, 56 °C for 45 s, and 72 °C for 60 s) were performed with a final extension at 72 °C for 7 min on a Bio-Rad C1000 thermal cycler. Triplicate PCR were performed per sample and then were pooled, purified using the QIAquick PCR Purification kit (QIAGEN), and quantified using a NanoDrop ND-1000 (Thermo Scientific, USA). The bar-coded PCR products from all samples were normalized in equimolar amounts to construct the library using a MiSeq Regent kit v3 (PE 2 × 300), and then were sequenced on an Illumina MiSeq platform in our institute (Illumina Inc., CA, USA).

Bacterial 16S rRNA gene sequence data were processed using the USEARCH pipeline. Raw paired-end (forward and reverse) sequences were merged with the “fastq_mergepairs” function and low-quality sequences (length < 200 or quality score < 20) were filtered with the “fastq_filter” function in USEARCH software [33]. We used the “fastx_uniques” function to perform the dereplication which could greatly reduce the computational memory demand. The functions “cluster_otus” and “unoise3” were then used to cluster unique sequences into operational taxonomic units (OTUs), and to perform error-correction on sequences (denoising into zero-radius OTUs, zOTUs), respectively. Singletons were discarded as they potentially result from erroneous sequencing or prediction. Phylotype identification was achieved using RDP classifier with a ≥ 80% probability threshold [34]. OTU table was constructed using the “otutab” function, and then combined with phylotype identification in R. We removed phylotypes that were not assigned as Bacteria and detected in less than 10 times across the data set. Finally, validated data were rarefied to 8200 (for 97% clustering) and 9500 sequences (for denoising) per sample to balance the sampling efforts, respectively. Beta diversity plots were generated with the rarefied data using the QIIME beta_diversity_through_plots.py script based on Bray Curtis metrics [35]. The two approaches produced considerably overlapping communities (21,112 phylotypes for 97% clustering and 29,184 phylotypes for denoising, respectively), with alpha and beta diversity metrics being highly positively correlated (r > 0.73 for observed richness, and r > 0.98 for Bray–Curtis dissimilarity, Additional file 1: Fig. S2). The denoising approach only increased (by 1.4 times) the resolution of bacterial diversity, in line with recent systematic investigations that demonstrated the limited effect of the OTU approach on the resulting microbial community structure [8]. Although the 97% similarity threshold is widely accepted to define molecular OTUs from the bacterial 16S rRNA region [4], the use of the denoised zOTUs has the important advantage at a much higher resolution of allowing for more precise identification down to the species level and even potentially beyond [8]. Therefore, given the substantial overlap between the two approaches, we decided to use the denoised zOTUs for all the downstream analyses. The datasets generated and analyzed during the current study are available in the National Center for Biotechnology Information (NCBI) repository under the accession number PRJNA726588.

### Validation for retrospective microbial analysis based on archived soil samples

We first estimated the fidelity of the archived soil samples for retrospect analysis according to their soil chemical characteristics (i.e., SOC, TN, TP, TK) and bacterial community composition via the Random Forest algorithm. This method has previously been shown to outperform other modeling approaches when used for environmental bacterial datasets [13, 36]. In these classification models (number of trees = 500, permutations = 999), fertilization strategy (a vector including seven factors that correspond to the seven fertilization treatments used) was set as response variable while soil chemical characteristics or community composition was set as predictor variable, in which fertilization time (in years from 0 to 20) were set as strata (i.e. a factor variable that is used for stratified sampling) to take into consideration the temporal dynamics in field conditions. The results showed that our archived soil samples were well stored to the extent that we could use the soil chemical properties (accuracy of 91.5% estimated via the out of bag error rate in Random Forest models) and bacterial community (accuracy of 86.73%) to accurately identify the fertilizer treatment the sample originated from (Additional file 1: Fig. S3). This high match accuracy could be comparable to those based on fresh samples [37], and provides high confidence in our results. Random Forest analysis was performed using the R package rfPermute (with default parameters, v2.1.81 [38]).

### Identifying the indicator microbiome and co-occurrence network construction

We first selected dominant taxa by retaining ubiquitous zOTUs that were present in more than 80% of all soil samples (n = 544), which yielded a subset community consisting of 604 phylotypes. We did not apply abundance criteria in this analysis because microbial keystone taxa might exert their influence (either individually or as a guild) on ecosystem functioning irrespective of their abundance across space and time (such as those involved in nitrogen fixation or ammonia oxidation) [5]. Ecological co-occurrence networks provide critical information on the potential associations among soil microorganisms and can be used to identify soil indicator phylotypes and/or consortia associated with the high levels of soil functioning and crop production [5, 23]. All possible Spearman rank correlations between OTUs across samples and corresponding *P* values were calculated. We corrected the false discovery rate according to Benjamini–Hochberg [39]. We considered a valid co-occurrence event to be robust if the Spearman correlation coefficient |ρ|> 0.6 and statistically significant at *P* < 0.01 [40, 41]. Pairwise Spearman correlation was calculated using the function ‘rcorr’ in the R package Hmisc (v4.4–0) [42], and the co-occurrence network was constructed using the function ‘graph.adjacency’ in the R package picante (v1.8.1). We also detected potential clusters/modules within cooccurrence network using the function ‘fastgreedy.community’ in the R picante package, as ecological clusters represent important ecological units that provide the opportunity to identify highly connected and indicator taxa [43]. An interactive platform “Gephi” (default parameters set, v0.9.1 [44]) was used to visualize the cooccurrence network. In the network graph, the nodes represent the individual phylotypes, with the colors denoting the ecological modules to which they belong, and the sizes of the nodes are proportional to their relative abundances in the community. Edges represent significant pairwise correlations with their width proportional to the coefficient. We calculated the relative abundance of each cluster by summarizing the relative abundances of all phylotypes therein. We are aware of the compositional nature of microbiome data [45] and of the fact that Spearman correlation is not a compositionally aware correlation metric [46]. When we tested with compositionally aware correlation metric, that is SparCC [47], despite the difference in network layout and scales, the outcomes and conclusions were essentially the same (Additional file 1: Fig. S4). In addition, Spearman approach generated a bigger-scale network whereby providing more potential candidates taxa that are fruitful target in management practices [4, 41]. Therefore, we decided to choose Spearman approach in downstream analysis.

### Identification of accurate taxonomy and microbial functional traits

Given that the denoised zOTUs allow for precise identification down to the species level [8], we further validated taxonomy information by blasting our phylotypes against the 16S rRNA database in NCBI. Result showed that, 163 out of the 604 selected phylotypes had 16S rRNA sequences that could be assigned with > 98.5% identity (similarity threshold for differentiating two species based on 16S rRNA gene sequence [48]) to a type specimen with a sequenced genome in the NCBI database. Then, the corresponding type specimen annotations were taken as the taxonomy of these phylotypes. Accounting for duplicate matches, these 163 phylotypes accounted for 140 separate type organisms. We matched those phylotypes at the species level with publicly available functional microorganisms related to nitrogen (nitrogen fixation/M00175, nitrification/M00528, denitrification/M00529, dissimilatory nitrate reduction/M00530, assimilatory nitrate reduction/M00531, complete nitrification/comammox/M00804), phosphorus (phosphonate and phosphinate metabolism/ko00440, alkaline phosphatase/M00126), and sulfur (assimilatory sulfate reduction/M00176, dissimilatory sulfate reduction/M00596, thiosulfate oxidation by SOX complex/M00595) metabolism from the KEGG database (last accessed on April 25, 2021). We calculated the module functional proportion by summarizing the proportion of successfully matched phylotypes within each module.

### Statistical analysis

The importance of these dominant taxa was estimated by calculating their count ratio as well as their relative abundance ratio. Pearson correlation between phylotype/module proportion and soil chemical characteristics was calculated using the function “rcorr” in the R package Hmisc (v4.4–0) [42]. Ordinary least square method was used to estimate the relationship between community (alpha and beta) diversities generated by 97% clustering and denoising approaches as well as the dissimilarity between the whole community and the subset community consisting of selected dominant phylotypes. ANOVA was used to test the significant difference in the proportion of each module among fertilizer treatments. We used the “aov” function in the R package Stats (v3.6.3) to conduct this analysis with the formula *aov(module%* ~ *fertilizer* + *Error (duration/fertilizer))*, in which fertilizer is the fixed variable, but experiment duration (years) is random and fertilizer is nested within duration to take into account of temporal variation in field conditions. The circular phylogenetic tree was visualized using the online tool iTOL [49]. All statistical analyses in this study were based on R version v3.6.3 [42].

## Results

### Fidelity of the archived soil samples for the retrospective analysis

We first checked the chemical fidelity of the archived soil samples. Based on their chemical signature we could accurately assign a certain sample to a certain treatment with > 91% confidence (Additional file 1: Fig. S3a). Then we performed the same approach based on the microbial community signatures of the samples, and the result was again very positive: we could identify with 86.73% certainty from what treatment series the (stored) sample was derived (Additional file 1: Fig. S3b).

**Fig. 1:**
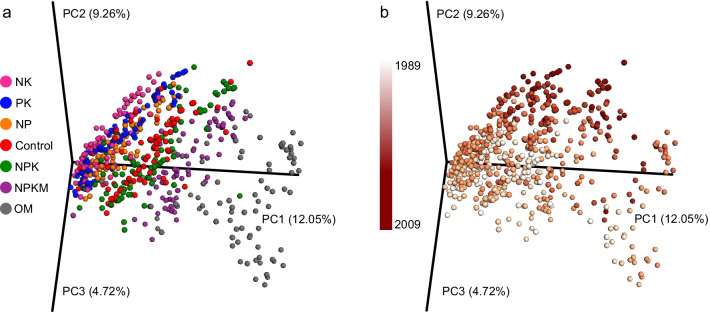
Fertilization type and duration strongly structure the microbial community. Projection of the data in PCoA based on Bray Curtis distance. Symbols represent microbiomes and are colored by fertilization type (**a** by the specific fertilization type of origin) by duration (**b** sampling year, color gradient). The first three PCs are plotted with the percentage of variation explained by each PC

Principal coordinate analysis (PCoA) based on Bray–Curtis distances showed that soil bacterial community exhibited distinct patterning according to the fertilization type (Fig. 1a), and to fertilization duration within each type (Fig. 1b). Strikingly, the samples formed four clusters based on fertilization types: two types of organic fertilization (OM and NPKM) were distinct from the chemical fertilizers and formed two independent clusters, whereas the three types of element-deficit fertilization (NP, NK, and PK) clustered together, while balanced chemical fertilizer (NPK) was more closely related to the no fertilized Control (Fig. 1a).

### Identification of the indicator phylotypes

**Fig. 2:**
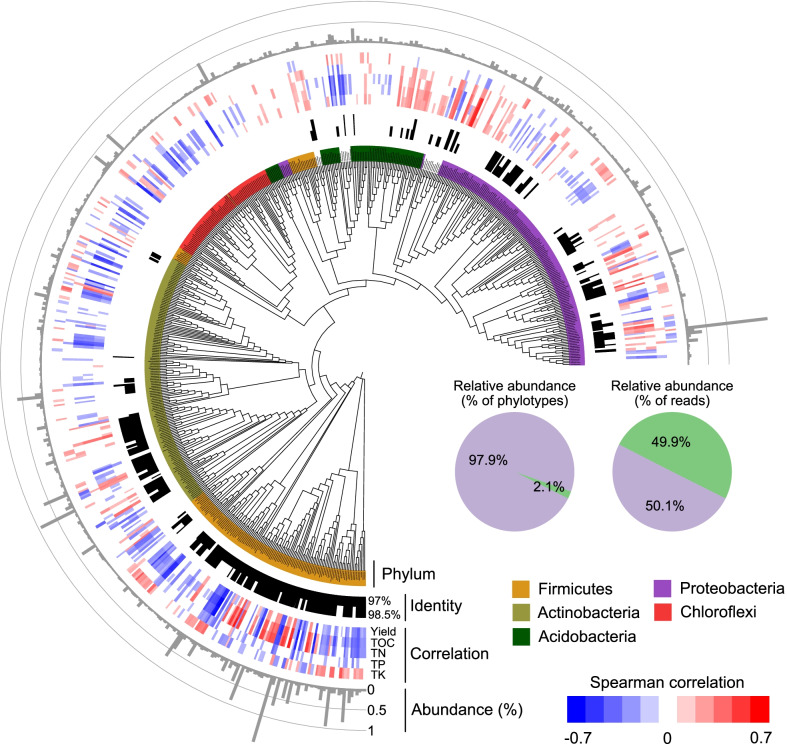
Identification of indicator microbiome in this temporal survey. The ratios of both abundance (%) and richness of the indicator microbiome phylotypes in the community are reported in the pie charts. The upset plot denotes the overlap of phylotypes between individual fertilizer-specific indicator microbiome. Phylogeny of indicator microbiome identified in this temporal survey (**d**). The tree shows the phylogenetic relationships of OTUs (n = 604) persistently present in individual fertilizer type. Ring “Phylum” indicates the most closely related bacterial type strain retrieved from GenBank, with sequence identity > 97% and 98.5% colored in black in adjacent ring “Identify”. Ring “Correlation” showed the spearman correlation coefficient between the proportion of individual phylotype and crop yield as well as multiple soil nutrient variables (TOC, TN, TP, TK). Ring “Abundance (%)” represent the proportion of individual phylotype in bacterial community. Blank cells in rings “Identity”, “Correlation” represent bacterial phylotypes that failed to match the threshold, i.e., sequence identity > 97% and 98.5% or statistical significance *P* < 0.05

As microbes that are consistently present with high frequency are likely to provide critical ecological functions, we searched for microbial taxa that are detected in more than 80% of samples. This streamlined microbial consortium consisted of 604 OTUs from a small set of bacterial phyla (i.e., phyla Proteobacteria, Firmicutes, Actinobacteria, Chloroflexi and Acidobacteria), which only represented ~ 2.1% of all the 29,184 sampled bacterial phylotypes, while contributing to nearly half of the total reads identified as Bacteria (Fig. 2 and Additional file 2: Table S2). The variation in Bray–Curtis dissimilarity index of this streamlined consortium was positively correlated with the diversity of the whole bacterial community (r = 0.95, *P* < 0.001, Additional file 1: Fig. S5a). Individual phylotype abundance and frequency were positively correlated, with relatively few phylotypes being both highly frequent and abundant, and the majority of the retrieved bacterial taxa restricted in their abundance (Additional file 1: Fig. S5b).

When compared against the GenBank database, 163 out of the 604 phylotypes had > 98.5% sequence identity with sequences obtained from publicly available bacterial type strains (Fig. 2 and Additional file 2: Table S2). The most abundant taxa included *Bacillus asahii* (2.05%), *Methylobacterium radiotolerans* (1.99%), *Priestia filamentosa* (0.94%) and *Blastococcus colisei* (0.93%). Importantly, majority members with their abundance were closely associated with multiple soil nutrient conditions as well as maize yield (Fig. 2), suggesting that this refined microbial consortium could well act as the indicator microbiome of soil fertility and crop production. Specifically, 40% of phylotypes affiliated with the phylum Firmicutes were negatively (*P* < 0.05) correlated with crop production, while more than one quarter (27%) of phylotypes in the phylum Proteobacteria (especially those in Alphaproteobacteria and in Gammaproteobacteria) were positively (*P* < 0.05) correlated with higher production (Additional file 2: Table S2). Nevertheless, several abundant phylotypes in the class Bacilli of phylum Firmicutes, such as *B. asahii* (r = 0.3), *Lysinibacillus sphaericus* (r = 0.31) were also positively correlated with higher production.

### Identification of the indicator microbiome

We used co-occurrence network analysis to identify phylotypes in the indicator microbiome that were highly connected with each other, and therefore, potentially shared environmental preferences and functional potentials (i.e., the effect on maize production in this study), which could go beyond predictions based on their taxonomic affiliations alone. This approach was feasible because the contrasting fertilization types supported different levels of maize production (Additional file 1: Fig. S1) and tended to give rise to distinct microbial communities (Fig. 1a).

**Fig. 3:**
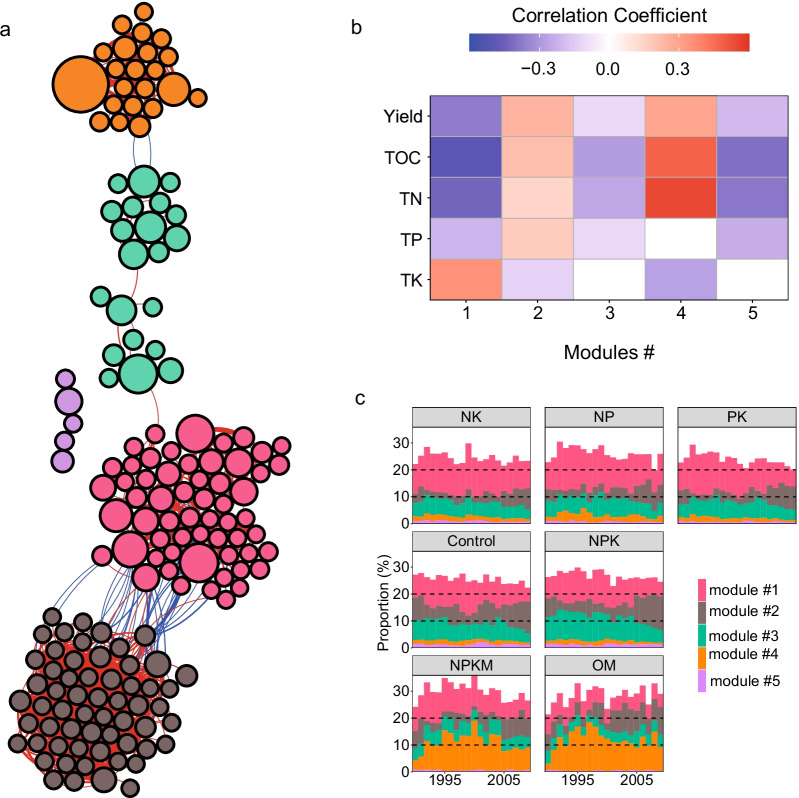
Co-occurrence network of indicator microbiome. Network diagrams with nodes (n = 165) colored according to different ecological modules. Nodes indicate bacterial phylotypes (OTUs) and edges represent significant co-occurrence relationships (Spearman’s ρ > 0.6 and *P* < 0.05). Node’s size corresponds to their proportion in the community. Edges colored in red or blue denote significant positive or negative correlation, respectively, and edge widths correspond the correlation coefficient values (**a**). The Pearson correlation between module abundance and soil physicochemical properties and maize production. Blank cells denote the non-significant correlation at the threshold of *P* < 0.05 (**b**). The dynamic abundance of contrasting modules in divergent fertilization types across the sampling duration (**c**)

We detected and identified several ecological clusters from the refined microbiome in the co-occurrence network. Many of the members (66 out of 165 phylotypes) of these clusters could be accurately identified at the species level based on the similarity threshold 98.5% (Fig. 2 and Additional file 2: Table S2). Specifically, *Cytobacillus firmus* (abundance = 0.88%), *Neobacillus mesonae* (0.81%) and *Bacillus anthracis* (0.56%) in phylum Firmicutes were the main members in module #1; module #2 was mainly comprised of *Nitrospira japonica* (0.27%) in phylum Nitrospirae and many other unclassified taxa in phyla Proteobacteria and Acidobacteria; module #3 was dominated by Actinobacteria, such as *Blastococcus colisei* (0.93%) and *Micrococcus terreus* (0.63%); and phylotypes in genus Bacillus of phylum Firmicutes dominated the module #4, in which *B*. *asahii* (1.98%) and *L*. *sphaericus* (0.31%) were the most abundant members (Fig. 3a). Members within each module were consistently and positively correlated with each other. Modules #1 and #2 were the main components in the network, and were connected through several phylotypes with negative correlations. Furthermore, while some members (e.g., *N*. *mesonae* and *B*. *anthracis* in module #1, *B*. *colisei* in module #3 and *B*. *asahii* in module #4) are important phylotypes that dominate the community and construct the module, dominant taxa are not necessarily guaranteed to be the keystone taxa in a community (e.g., *Priestia flexa* in module #1, *N*. *japonica* in module #2 and *L*. *sphaericus* in module #4) (Additional file 2: Table S2).

Network analysis revealed that the combined phylotype abundances of modules #2 and #4 were significantly and positively correlated with maize production and multiple soil nutrient contents (e.g., SOC and TN, TP), whereas those of modules #1, #3 and #5 exhibited opposite patterns (Fig. 3b). Furthermore, these two contrasting groups of modules also showed different distributional patterns across divergent fertilization types. Compared to the unfertilized control and chemical fertilizers, organic fertilization (e.g., OM and NPKM) consistently harbored a higher proportion of phylotypes in module #4 (*F* = 4.54, *P* < 0.001), while phylotypes in modules #1 (*F* = 2.78, *P* = 0.011) and #3 (*F* = 5.3, *P* < 0.001) were persistently rare in OM fertilization compared to chemical fertilization types (Fig. 3c).

### Multifunctionality of the indicator microbiome

**Fig. 4:**
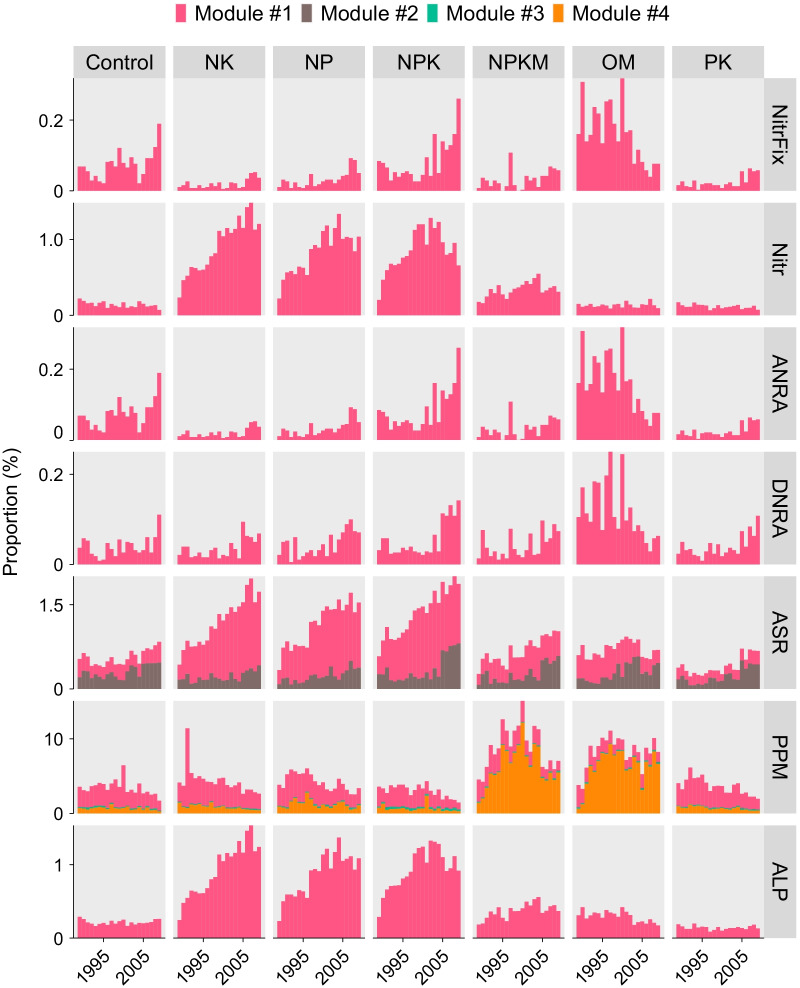
Multifunctionality of indicator microbiomes. We matched our bacterial phylotypes against the KEGG module table related to nitrogen, phosphorus and sulfate metabolism. *NitrFix* nitrogen fixation, *Nitr* nitrification, *DNRA/ARNA* dissimilatory/assimilatory nitrate reduction to ammonium, *ASR* assimilatory sulfate reduction, *PPM* phosphonate and phosphinate metabolism, *ALP* alkaline phosphatase

Bacterial phylotypes in the indicator microbiome exhibited various metabolic functions related to nitrogen, phosphorus and sulfate cycling, with most of the members capable of decomposing organic phosphorus via phosphonate and phosphonite metabolism (Fig. 4). Bacterial phylotypes in module #2 (*N*. *japonica*) was associated with assimilatory sulfate reduction, and those in module #4 (i.e., *L*. *sphaericus*, and *B*. *asahii*) were involved in phosphonate/phosphinate metabolism. Members in module #1 were suggested to play important roles in multiple functions: *B. anthracis*, *P*. *filamentosa* and *N*. *mesonae* combined play key roles in phosphonate and phosphinate metabolism, while *Nitrosospira multiformis* is involved in nitrification, assimilatory sulfate reduction and alkaline phosphatase production, and was enriched under chemical nitrogen fertilization, *Rhodopseudomonas palustris* is active in both nitrogen fixation and assimilatory nitrate reduction to ammonium (Fig. 4). Nitrogen fertilizer treatment exhibited a higher proportion of phylotypes in module #1 that were involved in nitrification (*F* = 47.59, *P* < 0.001) and alkaline phosphatase (*F* = 54.37, *P* < 0.001) as well as assimilatory sulfate reduction (*F* = 55.71, *P* < 0.001), while organic fertilizer samples showed higher potential in nitrogen fixation (*F* = 14, *P* < 0.001), nitrate reduction to ammonium (*F* = 14 for ANRA and *F* = 11.20 for DNRA, *P* < 0.001) in module #1 and phosphonate and phosphonite metabolism (*F* = 5.58, *P* < 0.001) in module #4.

## Discussion

### Functional traits of indicator phylotypes for soil fertility and crop yield

Harnessing microbiomes offers an invaluable approach to achieve agroecosystem sustainability with joint consideration of food security and environmental stress [6, 10]. In this study, we addressed this issue by characterizing the identity, fertilization-dependency and ecology of indictor microbiomes identified in a long-term (> 20 years) fertilization experiment conducted in the North China Plain that encompassed seven frequently applied fertilization types. To confidently detect and characterize the indicator microbiota, we selected those phylotypes that appeared at high frequency over 20 years. This stringent criterion generated a set of streamlined indicator phylotypes (~ 2.1%) from a few bacterial phyla that accounted for almost half (~ 50%) of the community population (Fig. 2a). These indicator phylotypes encompassed both dominant taxa with considerable abundance affecting broad processes (e.g., organic matter decomposition) and rare microbes exerting disproportional influences on specific well-defined processes (e.g., nitrogen fixation and ammonia oxidation) (Figs. 3a, 4). Many members of these indicator phylotypes are on record as indicator or keystone microbes in various agroecosystems, such as the most abundant taxa identified in this study like *B*. *asahii* and *M*. *radiotolerans* (Fig. 2d and Additional file 2: Table S2). As most bacteria generally do not take up macromolecules, Bacillus spp. naturally secrete a wide range of hydrolytic enzymes to decompose complex organic matter and to release mineral nutrients (such as phosphonate) into their environment, making them keystone taxa in the soil food-web [14, 50], and pioneers when exposed to organic matter amendment in alkaline soils [14, 51]. Indeed, *B*. *asahii* was found to dominate the bacterial community from 2 to 4 years onward under organic fertilization in our previous investigation [14]. In comparison, *M*. *radiotolerans* was more abundant under nutrient-deficit chemical fertilization types (Additional file 2: Table S2), helping the crop resist nutrient deficiencies by forming a symbiotic relationship with the plants in which *M*. *radiotolerans* consumes plant waste as a carbon source and in turn generates substances promoting plant growth [52].

With respect to the indicators of rare phylotypes, for example, *R*. *palustris* is a soil-borne phototroph that has been shown to exert positive effects on plant growth by stimulating nitrogen uptake and elevating auxin levels in expanding leaves [53]. *R*. *palustris* was enriched under OM treatment in this study (Additional file 2: Table S2), suggesting that organic fertilization supported a stronger soil nitrogen fixation ability. *N*. *multiformis* is an important nitrifier in upland soil and was enriched under chemical N fertilization (Additional file 2: Table S2), which might limit N use efficiency in upland soil by accelerating nitrification and denitrification [54]. Evidence of the consistent presence of such rare taxa with a potentially disproportional influence raises the possibility that members of the rare biosphere are nevertheless indispensable in agroecosystem functioning [5]. Overall, the close association between limited indicator phylotypes and specific fertilization-induced nutrient cycling across the two-decadal duration suggests the possibility and/or feasibility of employing microbes to achieve the desired results by altering farming practices.

### Multifunctionality of the indicator microbiome for soil fertility and crop yield

Ecological co-occurrence networks have been widely used to identify co-occurring microorganisms in the community and could potentially provide critical information on the multifunctionality of microbiomes [5, 23]. In this study, we identified several ecological modules comprised of the identified indicator phylotypes, in which members in each module positively correlated with each other (Fig. 3a). Importantly, these modules exhibited distinct ecosystem functions related to soil nutrients and carbon cycling (Fig. 4) and hence presented multifunctionality with soil fertility and crop yield (Fig. 3b, c). For example, module #1 was closely involved in biogeochemical processes such as nitrogen fixation, nitrification and organic phosphate decomposition (Fig. 4). N and P are the most limiting nutrients in terrestrial ecosystems; however, their availability for plants and soil biota largely depends on the depolymerization of soil organic matter and net mineralization by soil microorganisms [55]. Specifically, we found that OM treatment was associated with a higher abundance of phylotypes involved in nitrogen fixation (e.g., *R*. *palustris*) and nitrate reduction to ammonium, while chemical nitrogen fertilizer types enriched phylotypes active in nitrification (e.g., *N*. *multiformis*) and alkaline phosphatase (Fig. 4), suggesting competition of soil nutrients between soil microbes and plants under conventional chemical fertilization types [13]. *B*. *anthracis* in module #1 is a widely distributed soil-borne plant pathogen [56], and was found to be more enriched under nutrient-deficit fertilization (Additional file 2: Table S2), which might also account for the lower crop production in those farming systems. Accordingly, the microorganisms in module #1 were distributed in soils based on fertilization types, with their combined abundance negatively associated with soil fertility and crop yield (Fig. 3b).

In contrast, module #4 in the network exhibited solely and powerful functions in solubilizing phosphate by decomposing external organic matter (especially *B*. *asahii*), making it the most distinctive microbial signature for the sampling site in the North China Plain, where agricultural production is hindered by lower P availability [14]. In this context, amendment with external organic matter is necessary for promoting native microorganisms to increase the soil P availability, termed the coupled soil C and P cycling [51]. In addition, the external C-induced higher soil P availability paved the way for some beneficial phylotypes (i.e., *L*. *sphaericus* [57]) that could promote plant growth or control insects and pests. As a result, we found that microorganisms in module #4 were most abundant in OM treatments (Fig. 3c), and were closely associated with higher soil fertility and crop yield (Fig. 3b). Similarly, it has been reported that OM farming systems are characterized by specific microbial guilds known to be involved in the degradation of complex organic compounds, whereas systems receiving chemical fertilizer presumably harbor oligotrophic organisms adapted to nutrient-limited environments [13]. Importantly, long-term chemical fertilization could potentially suppress inherent soil nitrogen fixation by inhabiting nitrogen fixers [58]. Deploying microorganisms to increase plant nutrient uptake and resistance to biotic and abiotic stress has been postulated as one of the most promising long-term solutions to the integral challenges of achieving food security while supporting a healthy environment [6, 10]. Our results revealed that OM farming systems were more likely to accomplish this goal compared to conventional systems receiving chemical fertilizers in alkaline soils.

### Fidelity of the archived soil samples for the retrospective analysis

This study was based on archived soil samples collected from a long-term field experiment. We are aware that retrospective analysis of a microbial community based on archived soil samples poses a series of methodological and technical challenges. While archived samples are widely accepted for retrospective analysis of their chemical properties [59, 60], their application in retrospective microbiological studies remains controversial [61, 62]. In this study, we showed that the soil microbial communities were very well preserved to the extent that we could accurately assign, with high confidence (accuracy of 86.7%, Additional file 1: Fig. S3b) comparable to the confidence associated with fresh samples [37], a certain sample to a certain treatment based on their microbial signature. The microbial community showed a strong patterning of samples by fertilization type, with an obvious configuration according to the duration for each fertilization type (Fig. 1). Interestingly, across the duration of the field experiment, microbial communities under OM treatment were consistently separated from those that had developed under chemical fertilization and unfertilized control, confirming the frequently recorded distinct microbial diversity under conventional and OM farming systems at the time-series scale [13, 31, 63]. Strikingly, three types of unbalanced chemical fertilization with different element deficiencies harbored a similar microbial community composition, which was distinct from their balanced counterparts. The results based on soil chemical properties were even better (accuracy of 91.5%, Additional file 1: Fig. S3a). This high fidelity gives confidence that the microbial community in archived samples reflects the original community—while possibly not a 100% accurate reflection—to the extent that they can be used to identify indicator microbiome for soil fertility and crop yield in the sampling site on the Northern China Plain.

## Conclusions

In this study, we leveraged a retrospective analysis to investigate indicator microbiomes for soil fertility and crop yield in fields exposed to contrasting fertilization management practices. Using a stringent criterion, we identified an indicator microbiome consisting of limited but ubiquitous and abundant phylotypes, the majority of which were strongly associated with soil fertility and crop production. Furthermore, we identified two small subsets of coexisting members that exhibited opposite relationships with soil fertility and crop production in alkaline soils. One group consisted of members enriched under organic fertilization, which showed specific metabolic functions related to organic matter decomposition and was strongly associated with higher soil fertility and crop production at this type of sampling site. The other group encompassed microbial phylotypes that potentially limiting plant health and nutrient uptake and was found to be enriched under nutrient-deficit chemical fertilization practices. In summary, this study reports a “most-wanted” or “most-unwanted” list of microbial phylotypes that are feasible candidates for human manipulation in supporting crop production with explicit consideration of environmental sustainability.

## Supplementary Information

**Additional file 1 Supplementary information including yearly crop yield and additional statistical support for alpha and beta diversity measures: Figure S1**: Maize production of different fertilizer types across the duration; **Figure S2**. Comparison of community α(a) and β(b) diversities for the 97% clustering and sequence denoising approaches; **Figure S3**. Fidelity of archived samples in current study for retrospect analysis; **Figure S4**. Co-occurrence network of indicator microbiome based on SparCC correlation. **Figure S5**. The diversity of indicator microbial phylotypes.

**Additional file 2 Supplementary information including detailed experiment setup and indicator phylotypes: Table S1**. Detailed fertilization regimes in this study; **Table S2**. Detailed information of indicator phylotypes (n = 604).

## Data Availability

The datasets supported current study are available in the NCBI repository under the accession number PRJNA726588.
