# Supplementary material for: Beyond the snapshot: identification of the timeless, enduring indicator microbiome informing soil fertility and crop production in alkaline soils
Source: Environ Microbiome. 2022 May 12;17:25. doi: 10.1186/s40793-022-00420-6 (PMC9101894; doi:10.1186/s40793-022-00420-6)
Supplement: Supplementary file 1 — Additional file 1 Supplementary information including yearly crop yield and additional statistical support for alpha and beta diversity measures: Figure S1: Maize production of different fertilizer types across the duration; Figure S2. Comparison of community α(a) and β(b) diversities for the 97% clustering and sequence denoising approaches; Figure S3. Fidelity of archived samples in current study for retrospect analysis; Figure S4. Co-occurrence network of indicator microbiome based on SparCC correlation. Figure S5. The diversity of indicator microbial phylotypes. [file 40793_2022_420_MOESM1_ESM.docx]

**Beyond the snapshot: Identification of the timeless, enduring indicator microbiome informing soil fertility and crop production in alkaline soils**

Jianwei Zhang^a,b^, Jan Dolfing^c^, Wenjing Liu^a,b^, Ruirui Chen^a^, Jiabao Zhang^a^,

Xiangui Lin^a^, Youzhi Feng^a*^

^a^State Key Laboratory of Soil and Sustainable Agriculture, Institute of Soil Science, Chinese Academy of Sciences, Nanjing, 210008, PR China

^b^University of Chinese Academy of Sciences, Beijing, 100049, PR China

^c^Faculty of Engineering and Environment, Northumbria University, Newcastle upon Tyne, UK

Corresponding author^*^

Youzhi Feng, Email: [yzfeng@issas.ac.cn](mailto:yzfeng@issas.ac.cn)

**Supplementary Information**


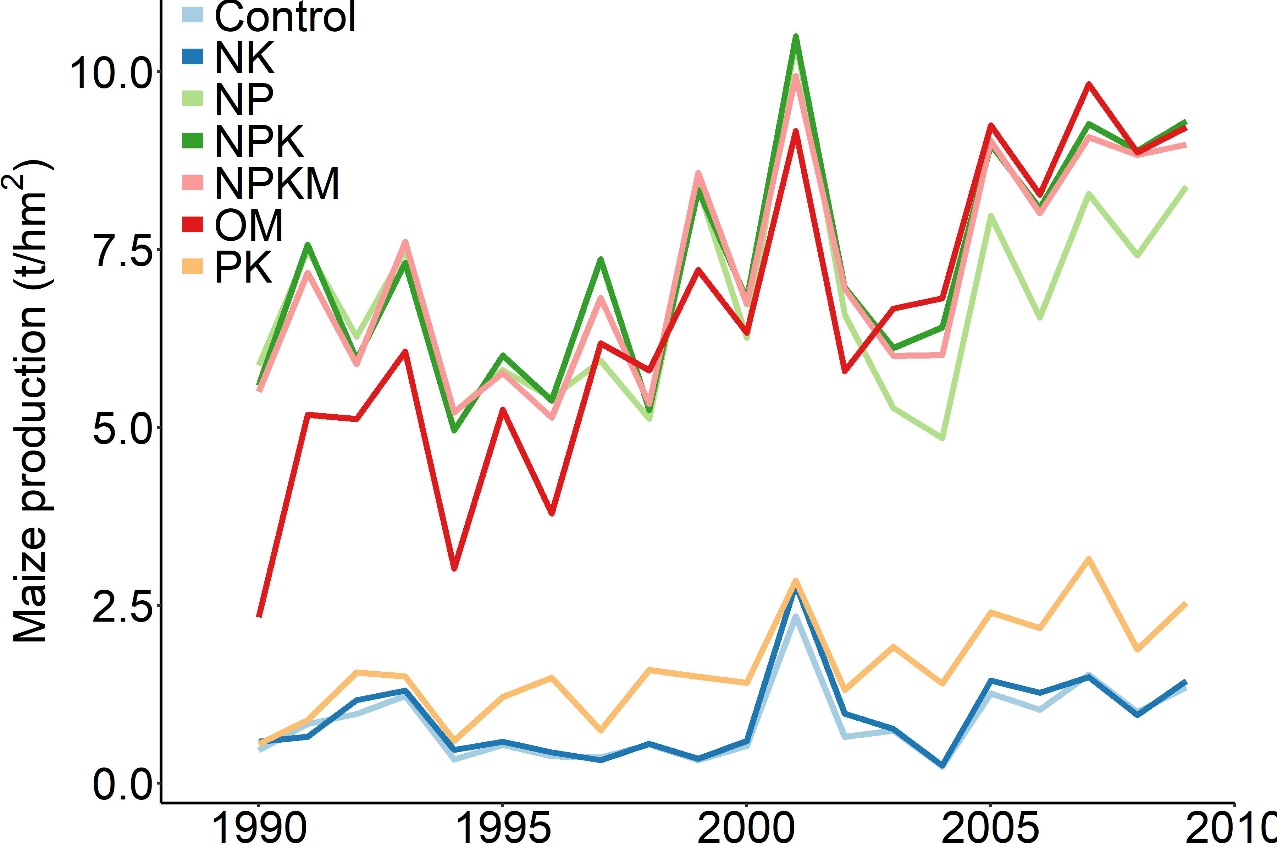


**Figure S1.** **Maize production of different fertilizer types across the duration.** We do not measure maize production in 1989 when the experiment began.


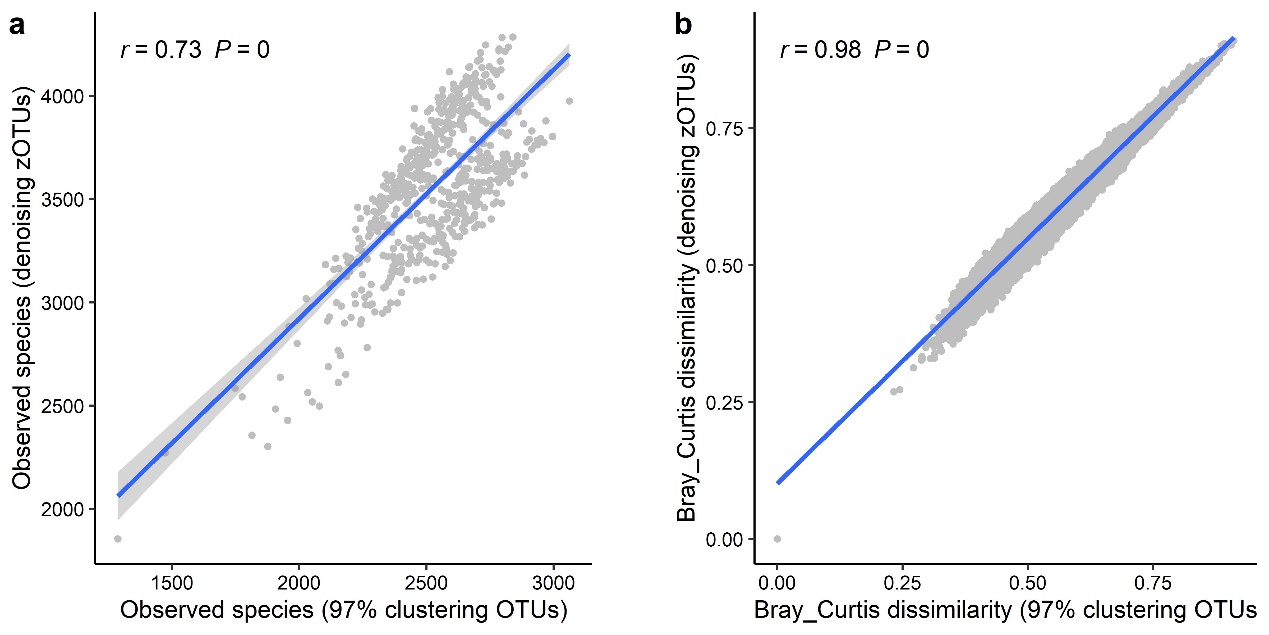


**Figure S2.** **Comparison of community α (a) and β (b) diversities for the 97% clustering and sequence denoising approaches**. Statistical coefficients are labeled on the panel. Shaded areas represent confidence intervals (95%).

**Figure S3.** **Fidelity of archived samples in current study for retrospect analysis**. We leveraged Random Forest classification algorithm to predict the origin treatments to which a sample belong based on soil nutrient contents (e.g., SOC, TN, TP, TK) (a) and microbial community composition (b). Numbers in the diagonal indicate correct classifications.


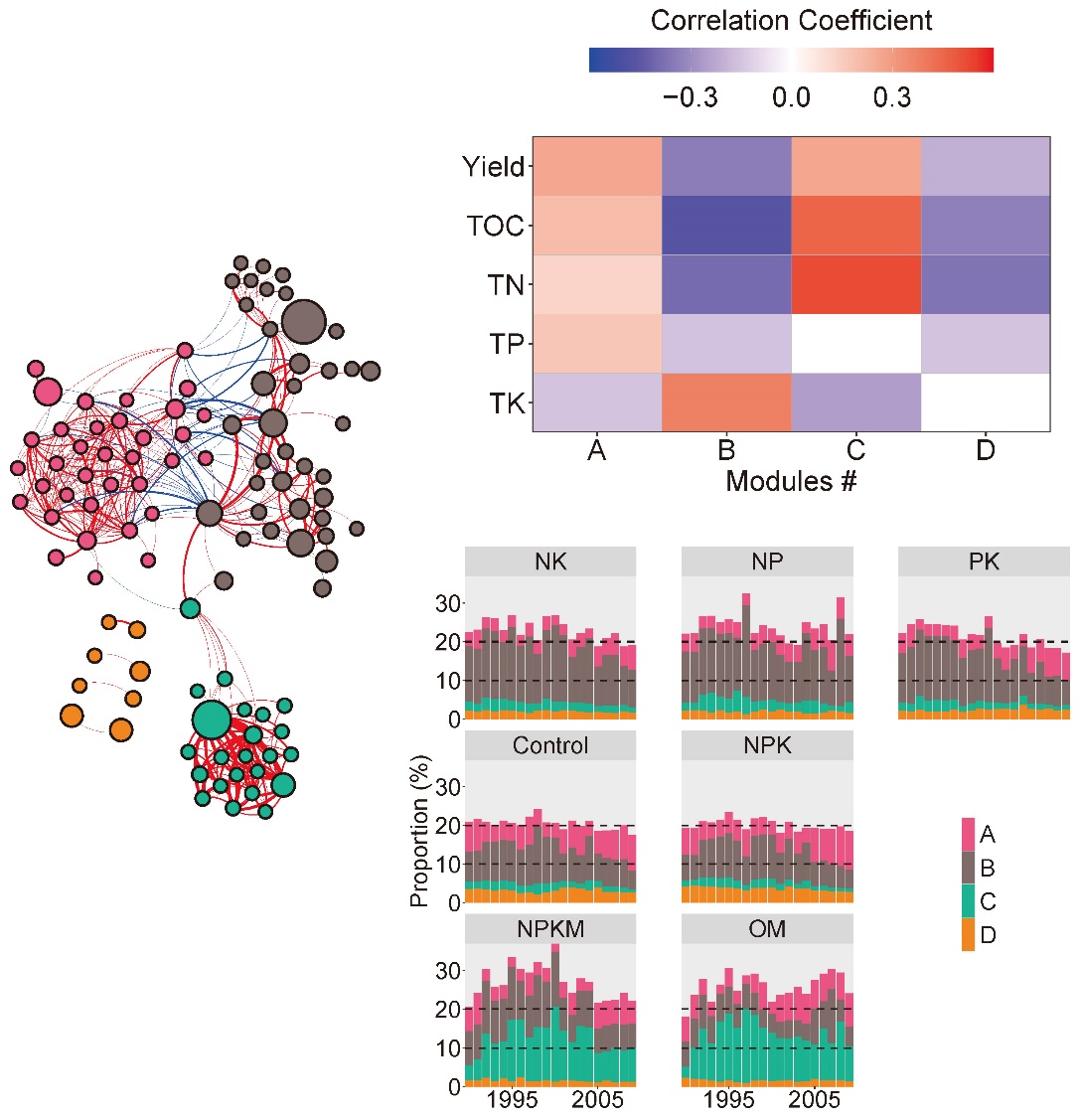


**Fig. S4.** **Co-occurrence network of indicator microbiome based on SparCC correlation.** Network diagrams with nodes (n = 105) colored according to different ecological modules. Nodes indicate bacterial phylotypes (OTUs) and edges represent significant co-occurrence relationships (r > 0.6 and P < 0.05). Node’s size corresponds to their proportion in the community. Edges colored in red or blue denote significant positive or negative correlation, respectively, and edge widths correspond the correlation coefficient values. The heatmap showed the Pearson correlation between module abundance and soil physicochemical properties and maize production. Blank cells denote the non-significant correlation at the threshold of P < 0.05. The bar charts denoted the dynamic abundance of contrasting modules in divergent fertilization types across the sampling duration.


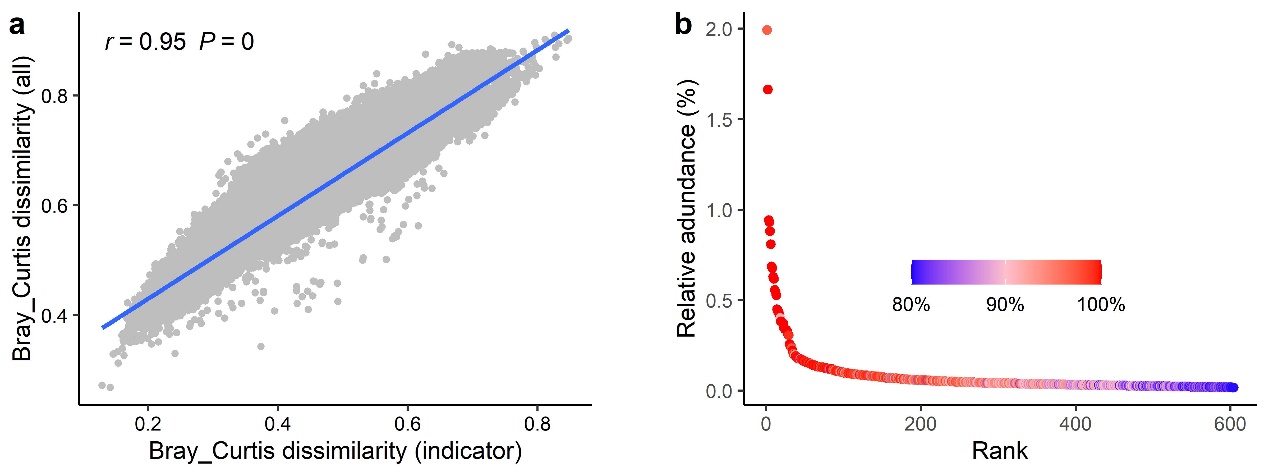


**Figure S5.** **The diversity of indicator microbial phylotypes.** Comparison of β diversity (Bray-Curtis dissimilarity index) for indicator phylotypes and whole bacterial community (a). Rank abundance curve for indicator phylotypes, with the phylotypes ordered on the x-axis from most to least abundant, and colors from blue to red corresponds to number of sites occupied (b).
